# Supplementary material for: Soluble Hemojuvelin and Ferritin: Potential Prognostic Markers in Pediatric Hematopoietic Cell Transplantation
Source: Cancers (Basel). 2023 Feb 7;15(4):1041. doi: 10.3390/cancers15041041 (PMC9954506; doi:10.3390/cancers15041041)
Supplement: Supplementary file 1 [file cancers-15-01041-s001.zip › cancers-2161541-SI.pdf]

cancers

Supplementary material

Soluble Hemojuvelin and Ferritin: Potential Prognostic Markers in Pediatric Hematopoietic Cell Transplantation

Supplementary Table S1. Differences between parameters of iron metabolism.

**Parameters**	**Total number of patients**	**Controls (group I)**	**Acute leukemia at diagnosis (group II)**	**Acute leukemia after intensive chemotherapy (group III)**	**After HCT (group IV)**	**p-value**
n	137	**19**	**36**	**50**	**32**	
PRBC transfusions [units] median (range)	7 (0-99)	0 (0-0)	1 (0-10)	10 (2-40)	23 (5-99)	I vs II; p<0.001<br>I vs III; p<0.001<br>I vs IV; p<0.001<br>II vs III; p<0.001<br>II vs IV; p<0.001<br>III vs IV; p<0.001
Serum iron [mg/dL] median (range)	117.65 (10.0-265.4)	67.90 (20.1-97.4)	135.00 (39.0-262.0)	107.90 (10.0-264.0)	130.80 (41.9-265.4)	I vs II; p<0.001<br>I vs III; p<0.001<br>I vs IV; p<0.001<br>II vs III; p=0.084<br>II vs IV; p=0.530<br>III vs IV; p=0.347
Transferrin [mg/L] median (range)	852.8 (131.4-15000.0)	647.0 (218.8-15000.0)	860.0 (151.9-15000.0)	1205.9 (243.2-5206.0)	722.1 (131.4-2634.0)	I vs II; p=0.276<br>I vs III; p=0.015<br>I vs IV; p=0.953<br>II vs III; p=0.041<br>II vs IV; p=0.320<br>III vs IV; p=0.003
TIBC [µg/L] median (range)	267 (125-439)	355 (292-439)	269 (125-329)	267 (183-379)	242 (128-286)	I vs II; p<0.001<br>I vs III; p<0.001<br>I vs IV; p<0.001<br>II vs III; p=0.634<br>II vs IV; p=0.002<br>III vs IV; p<0.001
Ferritin [µg/L] median (range)	701.0 (5.4-12000.0)	26.9 (5.4-73.3)	325.6 (14.2-1660.0)	963.5 (22.0-5503.2)	3370.0 (1070.0-12000.0)	I vs II; p<0.001<br>I vs III; p<0.001<br>I vs IV; p<0.001

						II vs III; p=0.006<br>II vs IV; p<0.001<br>III vs IV; p<0.001
FTH1 [pg/mL]<br>median (range)	22.22<br>(0.00-726.50)	16.45<br>(0.53-83.87)	23.04<br>(0.00-<br>726.50)	24.59<br>(0.50-132.00)	21.95<br>(0.00-309.40)	I vs II; p=0.348<br>I vs III; p=0.207<br>I vs IV; p=0.938<br>II vs III; p=0.999<br>II vs IV; p=0.446<br>III vs IV; p=0.314
FTL [pg/mL]<br>median (range)	101.90<br>(0.00-571.80)	92.21<br>(29.44-571.80)	116.55<br>(43.29-<br>363.00)	102.95<br>(0.00--286.10)	98.32<br>(0.00-288.10)	I vs II; p=0.479<br>I vs III; p=0.582<br>I vs IV; p=0.838<br>II vs III; p=0.115<br>II vs IV; p=0.233<br>III vs IV; p=0.857
Hepcidin<br>[ng/mL]<br>median (range)	202.80<br>(9.63-1000.00)	30.61<br>(9.63-468.20)	229.20<br>(13.94-<br>738.60)	136.65<br>(17.26-734.90)	281.85<br>(27.99--1000.00)	I vs II; p<0.001<br>I vs III; p<0.001<br>I vs IV; p<0.001<br>II vs III; p=0.073<br>II vs IV; p=0.026<br>III vs IV; p<0.001
sHJV [ng/mL]<br>median (range)	46.89<br>(18.34-136.80)	63.47<br>(28.78-91.47)	45.72<br>(27.33-<br>88.23)	46.23<br>(24.78-136.80)	38.97<br>(18.34-98.41)	I vs II; p<0.001<br>I vs III; p<0.001<br>I vs IV; p<0.001<br>II vs III; p=0.969<br>II vs IV; p=0.020<br>III vs IV; p=0.012
Ferritin / sHJV<br>ratio<br>median (range)	15.01<br>(0.19-341.27)	0.31<br>(0.19-0.87)	8.05<br>(0.25-33.44)	19.71<br>(0.34-160.91)	91.76<br>(11.18-341.27)	I vs II; p<0.001<br>I vs III; p<0.001<br>I vs IV; p<0.001<br>II vs III; p=0.012<br>II vs IV; p<0.001<br>III vs IV; p<0.001
FNP [pg/mL]<br>median (range)	84.72<br>(18.42-1321.00)	75.75<br>(18.42-251.90)	84.92<br>(29.12-<br>924.70)	86.08<br>(38.57-1321.00)	114.35<br>(27.11-1250.00)	I vs II; p=0.697<br>I vs III; p=0.768<br>I vs IV; p=0.102<br>II vs III; p=0.999<br>II vs IV; p=0.219<br>III vs IV; p=0.233
Erythroferrone<br>(ERFE) [ng/mL]<br>median (range)	8.482<br>(1.413-100.0)	8.236<br>(2.972-100.0)	10.201<br>(2.332-<br>100.0)	7.825<br>(1.413-100.0)	7.856<br>(1.831-100.0)	I vs II; p=0.371<br>I vs III; p=0.662<br>I vs IV; p=0.477

						II vs III; p=0.108<br>II vs IV; p=0.112<br>III vs IV; p=0.686
EPO [mIU/mL]<br>median (range)	30.62<br>(0.90-200.0)	10.88<br>(0.90-25.46)	85.39<br>(3.62-200.0)	30.92<br>(8.59-200.0)	24.31<br>(6.20-200.0)	I vs II; p<0.001<br>I vs III; p<0.001<br>I vs IV; p<0.001<br>II vs III; p<0.001<br>II vs IV; p<0.001<br>III vs IV; p=0.035
sTfR [µg/mL]<br>median (range)	0.613<br>(0.358-6.285)	0.608<br>(0.377-1.866)	0.606<br>(0.380-<br>6.285)	0.839<br>(0.362-3.527)	0.507<br>(0.358-3.192)	I vs II; p=0.846<br>I vs III; p=0.498<br>I vs IV; p=0.471<br>II vs III; p=0.730<br>II vs IV; p=0.219<br>III vs IV; p=0.133
CRP [mg/L]<br>median (range)	0.90<br>(0.05-297.54)	0.47<br>(0.16-2.34)	1.03<br>(0.37-<br>156.50)	0.50<br>(0.05-36.37)	3.27<br>(0.22-297.54)	I vs II; p=0.004<br>I vs III; p=0.488<br>I vs IV; p<0.001<br>II vs III; p<0.001<br>II vs IV; p=0.381<br>III vs IV; p<0.001
PCT [ng/mL]<br>median (range)	0.06<br>(0.00-11.47)	0.02<br>(0.02-0.10)	0.08<br>(0.01-2.82)	0.05<br>(0.00-1.20)	0.12<br>(0.02-11.47)	I vs II; p<0.001<br>I vs III; p<0.001<br>I vs IV; p<0.001<br>II vs III; p<0.001<br>II vs IV; p=0.651<br>III vs IV; p<0.001

Abbreviations: sHJV, soluble hemojuvelin; sFNP-1, soluble ferroportin-1; ERFE, erythroferrone; EPO, erythropoietin; sTfR, soluble transferrin receptor; TIBC, total iron-binding capacity; FTH1, ferritin heavy chain; FTL, ferritin light chain; CRP, C-reactive protein; PCT, procalcitonin; HCT, hematopoietic cell transplantation; PRBC, packed red blood cell concentrate.
